# Transcriptomic profiling of wheat near-isogenic lines reveals candidate genes on chromosome 3A for pre-harvest sprouting resistance

**DOI:** 10.1186/s12870-021-02824-x

**Published:** 2021-01-21

**Authors:** Xingyi Wang, Hui Liu, Kadambot H. M. Siddique, Guijun Yan

**Affiliations:** 1 UWA School of Agriculture and Environment, The University of Western Australia, Perth, WA 6009 Australia; 2 The UWA Institute of Agriculture, The University of Western Australia, Perth, WA 6009 Australia

**Keywords:** RNA sequencing, Wheat, Pre-harvest sprouting, Marker-assisted selection, Near-isogenic lines

## Abstract

**Background:**

Pre-harvest sprouting (PHS) in wheat can cause severe damage to both grain yield and quality. Resistance to PHS is a quantitative trait controlled by many genes located across all 21 wheat chromosomes. The study targeted a large-effect quantitative trait locus (QTL) *QPhs.ccsu-3A.1* for PHS resistance using several sets previously developed near-isogenic lines (NILs). Two pairs of NILs with highly significant phenotypic differences between the isolines were examined by RNA sequencing for their transcriptomic profiles on developing seeds at 15, 25 and 35 days after pollination (DAP) to identify candidate genes underlying the QTL and elucidate gene effects on PHS resistance. At each DAP, differentially expressed genes (DEGs) between the isolines were investigated.

**Results:**

Gene ontology and KEGG pathway enrichment analyses of key DEGs suggested that six candidate genes underlie *QPhs.ccsu-3A.1* responsible for PHS resistance in wheat. Candidate gene expression was further validated by quantitative RT-PCR. Within the targeted QTL interval, 16 genetic variants including five single nucleotide polymorphisms (SNPs) and 11 indels showed consistent polymorphism between resistant and susceptible isolines.

**Conclusions:**

The targeted QTL is confirmed to harbor core genes related to hormone signaling pathways that can be exploited as a key genomic region for marker-assisted selection. The candidate genes and SNP/indel markers detected in this study are valuable resources for understanding the mechanism of PHS resistance and for marker-assisted breeding of the trait in wheat.

**Supplementary Information:**

The online version contains supplementary material available at 10.1186/s12870-021-02824-x.

## Background

Wheat (*Triticum aestivum* L.) is a major cereal crop worldwide. Pre-harvest sprouting (PHS) can severely affect to yield and its nutritional and processing qualities, resulting in more than US$ 1 billion of annual losses worldwide [1, 2]. Therefore, PHS resistance is an important trait for genetic studies and breeding in wheat [3, 4].

Seed dormancy and germination, the two major processes concerning PHS, are regulated by numerous environmental and molecular factors; of which, endogenous hormone balance, especially between abscisic acid (ABA) and gibberellic acid (GA), plays a crucial role [5, 6]. In cereal grains, ABA is involved in dormancy development and inhibition of hydrolase synthesis in mature seeds [7], whereas GA promotes the metabolism of seed reserves and induces hydrolase synthesis for seed germination [8]. Apart from phytohormone transduction genes, many transcription factors (TFs) are involved in PHS regulation, such as those of the B3 domain, AP2 domain, and bZIP factor classes encoded by ABA-insensitive (ABI) genes *ABI3*, *ABI4*, and *ABI5*, respectively [9, 10], TFIIS Transcription Elongation Factor II encoded by *Reduced Dormancy 2* (*RDO2*) [11], and phytochrome interacting factors (PIFs) [12].

**Fig. 1:**
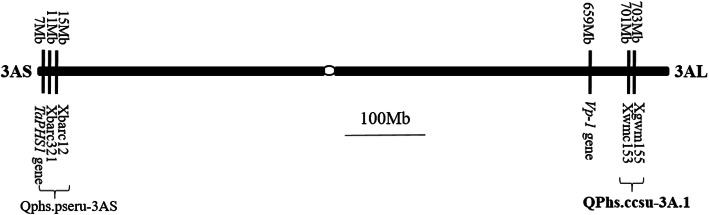
Diagram showing the physical locations of cloned genes and flanking markers of major QTL on chromosome 3A for seed dormancy and preharvest sprouting resistance. QTL in bold is the targeted QTL in this study. Bar represents 100 Mb physical distance

Resistance to PHS is controlled by quantitative trait loci (QTL) [13–15] that are located on all 21 chromosomes in bread wheat; of which, QTL on chromosome groups 3 and 4 consistently explain large phenotypic variation [16–19]. Several candidate genes have been identified for a major 4AL locus responsible for PHS resistance, including two seed dormancy genes *PM19-A1* and *A2* by transcriptomic analyses [20], and a causal seed dormancy gene *MKK3* located next to *PM19* by a comparative genomics method [21]. Wang et al. [22] identified five candidate genes for a major 4BL QTL using genotyping and phenotyping characterization of multiple pairs of near isogenic lines (NILs). For group 3 chromosomes, a major locus on 3AS, explaining 23–38% of the phenotypic variation, was identified using a cross-derived RIL population with red-grained parents [23]. Later, Liu et al. [24] cloned a gene (*TaPHS1*) from the 3AS QTL *Qphs.pseru-3AS*. Other known genes on group 3 chromosomes include *viviparous* (*Vp-1*) or *ABI3* [25] on the long arms of the chromosomes, which act as a regulator of late embryo development in wheat. Kulwal et al. [26] reported a major PHS resistance QTL on 3AL from RILs of SPR8198 (PHS resistant) / HD2329 (PHS susceptible). *QPhs.ccsu-3A.1* explained up to 78.03% of the phenotypic variation across six tested environments, and was located at a genetic distance of ~ 183 cM from the centromere within the marker interval of Xwmc153 and Xgwm155 [26]. This major QTL on chromosome arm 3AL has not been cloned and characterized (Fig. 1).

In our previous study, we developed several sets of resistant and susceptible NILs targeting the major QTL *QPhs.ccsu-3A.1* [27]. Near isogenic lines (NILs) are pairs of lines that have the same genetic background except for the targeted locus, and NILs with contrasting trait performance can turn quantitative traits into Mendelian factors, which makes them ideal genetic resources for identifying candidate genes and closely linked markers underlying a targeting QTL [22, 27]. RNA sequencing (RNA-seq) is a powerful approach for detecting differentially expressed genes (DEGs) and novel expressed genes, and is widely used in transcriptomic studies [28–30]. The expression trends of all genes from the transcriptomic analysis will be valuable data for in-depth studies of gene function and their interaction networks in complex biological processes [31]. Transcriptomic profiling of contrasting genotypes can reveal associated signaling pathways for molecular responses resulting in biochemical and morphological changes under stresses [29, 30]. Furthermore, RNA-seq on NILs can accurately detect DEGs and QTL-linked single nucleotide polymorphisms (SNPs) within a QTL region, therefore it has been used to identify candidate genes and markers in many crops [20, 32].

In this study, we used two pairs of NILs with highly significant differences in PHS performance between the isolines to investigate their transcriptomic profiles on developing seeds at 15, 25 and 35 days after pollination (DAP). The parental lines Chara and DM5637B*8 used to develop the NILs were white-coloured cultivars, which eliminated the possibility of correlations between PHS resistance and red-grain genes. The study aimed to: 1) analyze DEGs between the NILs at different seed development stages to provide an insight into PHS resistance, 2) validate the candidate genes through expression analysis at different seed developmental stages, and 3) detect SNPs and indels that can distinguish the resistant and susceptible isolines within the QTL interval for use in marker-assisted breeding of PHS resistance in wheat.

## Results

### Transcriptome assembly quality and mapping statistics

A total of 304 Gb high-quality 150-bp paired-end sequencing reads were generated from the 36 samples after quality control, with an average of 56 million clean reads for each library. Nearly 98 and 96% of the clean reads had a quality score of Q20 and Q30, respectively. Approximately 70% of the sequenced reads were mapped to the wheat reference genome, including 55% with a unique match. The total number of transcripts detected in each library ranged from 72,485 to 96,979, accounting for nearly 60% of all wheat genes. Pearson’s correlation coefficients among the three biological replicates for each combination ranged from 0.84 to 0.99, indicating the consistency of the three replicates.

### Differential gene expression related to PHS resistance

**Fig. 2:**
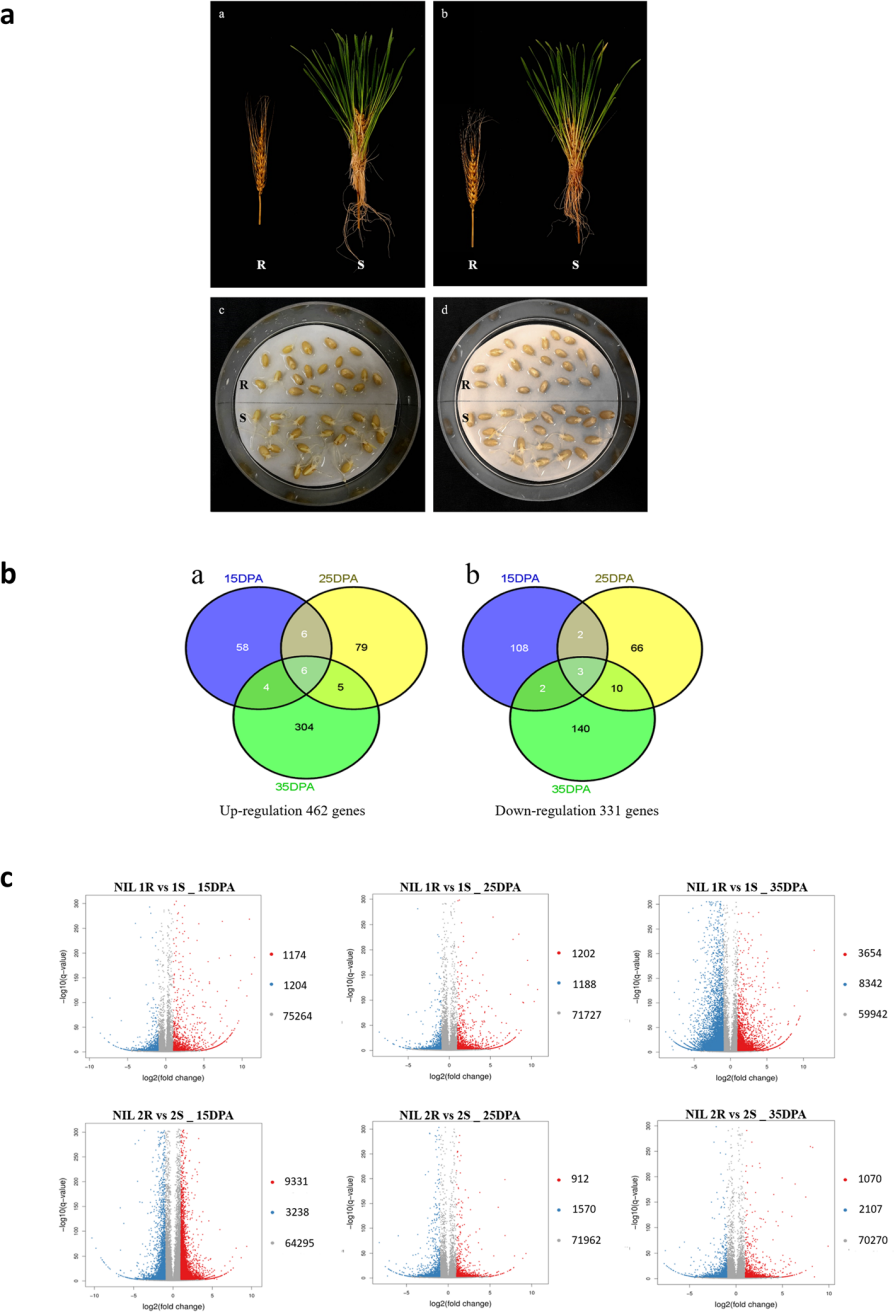
Comparison of NIL pairs 1 and 2. A. Phenotypic differences between resistant (R) and susceptible (S) isolines in the two NIL pairs. (a) and (b) were the spike sprouting test of NIL pair 1 (left) and NIL pair 2 (right), respectively, at day 7 of the test; (c) and (d) were seed germination test at day 2 of the test. B. Venn diagrams showing the number of differentially expressed genes (DEGs) that were commonly (a) up-regulated and (b) down-regulated in the resistant isolines compared with those in the susceptible isolines. Numerals inside the parentheses indicate the number of genes expressed at each time point. The total number of DEGs is noted at the bottom of each Venn diagram. C. Volcano plot showing DEGs within each NIL pair at different time-points. X axis represents log2 transformed fold change. Y axis represents -log10 transformed *p* value significance. Blue points represent up-regulated DEGs. Red points represent down-regulated DEGs. Gray points represent non-DEGs. DPA = days post anthesis

Differential gene expressions in the contrasting isolines are summarized in Table 1 and Fig. 2. At 15 DPA, a total of 1195 DEGs between the resistant (‘R’) and susceptible (‘S’) isolines were commonly detected in the two NIL pairs. Similar numbers (1298) of DEGs between the isolines were detected at 35 DPA. However, fewer DEGs (776) were detected at 25 DPA in both of the NIL pairs. To identify the genes underlying the QTL *QPhs.ccsu-3A.1*, particular attention was given to the common DEGs located on chromosome arm 3AL in both of the NIL pairs. There were 12, 12 and 25 such genes identified at 15, 25, and 35 DPA, respectively. Among them, genes *TraesCS3A01G462000* and *TraesCS3A01461400* were consistently upregulated (gene expression in ‘R’ isoline was significantly higher than that in ‘S’ isoline, i.e. ‘R>S’), and gene *TraesCS3A01G466700* was consistently downregulated (gene expression in ‘R’ isoline was significantly lower than that in ‘S’ isoline, i.e. ‘R<S’) across the three time-points. The three genes were located within the targeted QTL marker interval between Xwmc153 and Xgwm155, were therefore considered major candidate genes underlying *QPhs.ccsu-3A.1.* Notably, at all three time-points, *TraesCS3A01G461400* and *TraesCS3A01G466700* showed extremely high upregulation and downregulation, with a mean log2 ratio fold change of 5.70 and 5.19, respectively (Table S1).

**Table 1:** The number of differentially expressed genes across the whole genome and on chromosome 3AL between the resistant and susceptible isolines in the two NIL pairs

Time point	DEG	NIL pairs				Common	
		NIL pair 1		NIL pair 2			
		Genome	3AL	Genome	3AL	Genome	3AL
15DPA	Up	1204	29	3238	89	681	6
	Down	1174	12	9331	294	514	6
	Total	2378	41	12,569	383	1195	12
25DPA	Up	1188	26	1570	52	304	5
	Down	1202	23	912	26	472	7
	Total	2390	49	2482	78	776	12
35DPA	Up	8342	246	2107	76	715	14
	Down	3654	95	1070	42	583	11
	Total	11,996	341	3177	118	1298	25

*DPA* Days post anthesis, *DEG* Differentially expressed genes, *Up* (upregulated) and *Down* (downregulated), relative to susceptible isolines, *Common* DEGs common to both NIL pairs

Gene expressions significantly different between time-points in each isoline, including DEGs between 25 DPA and 15 DPA (25/15), DEGs between 35 DPA and 25 DPA (35/25), and DEGs between 35 DPA and 15 DPA (35/15), were investigated, especially for those within the 3AL QTL interval. A special focus was put on DEGs that showed noteworthy features, including those associated with hormone transduction and PHS-regulatory TFs (such as bZIP TFs, and B3 or AP2 domain-containing TFs), those associated with the identified SNP and indel variants, and those displaying significant differences between isolines at different time-points. Interestingly, *TraesCS3A01G461400* showed down-regulation in the ‘R’ isolines at 35/15, but it had significantly higher expression in the ‘R’ isolines than the ‘S’ isolines of both NIL pairs at 35 DPA. Other genes that shared the same up- or down- regulations in either ‘R’ or ‘S’ isolines at 35/15 included *TraesCS3A01G459200* (down-regulated in ‘R’ isolines, and ‘R>S’ at 15 and 25 DPA), *TraesCS3A01G470400* (down-regulated in ‘S’ isolines, and ‘R<S’ at 15 and 25 DPA), *TraesCS3A01G416200* (up-regulated in ‘R’ isolines, and ‘R>S’ at 35 DPA), and *TraesCS3A01G346700* (up-regulated in ‘S’ isolines, and ‘R<S’ at 35 DPA) (Table S1).

### Functional annotation of DEGs

**Fig. 3:**
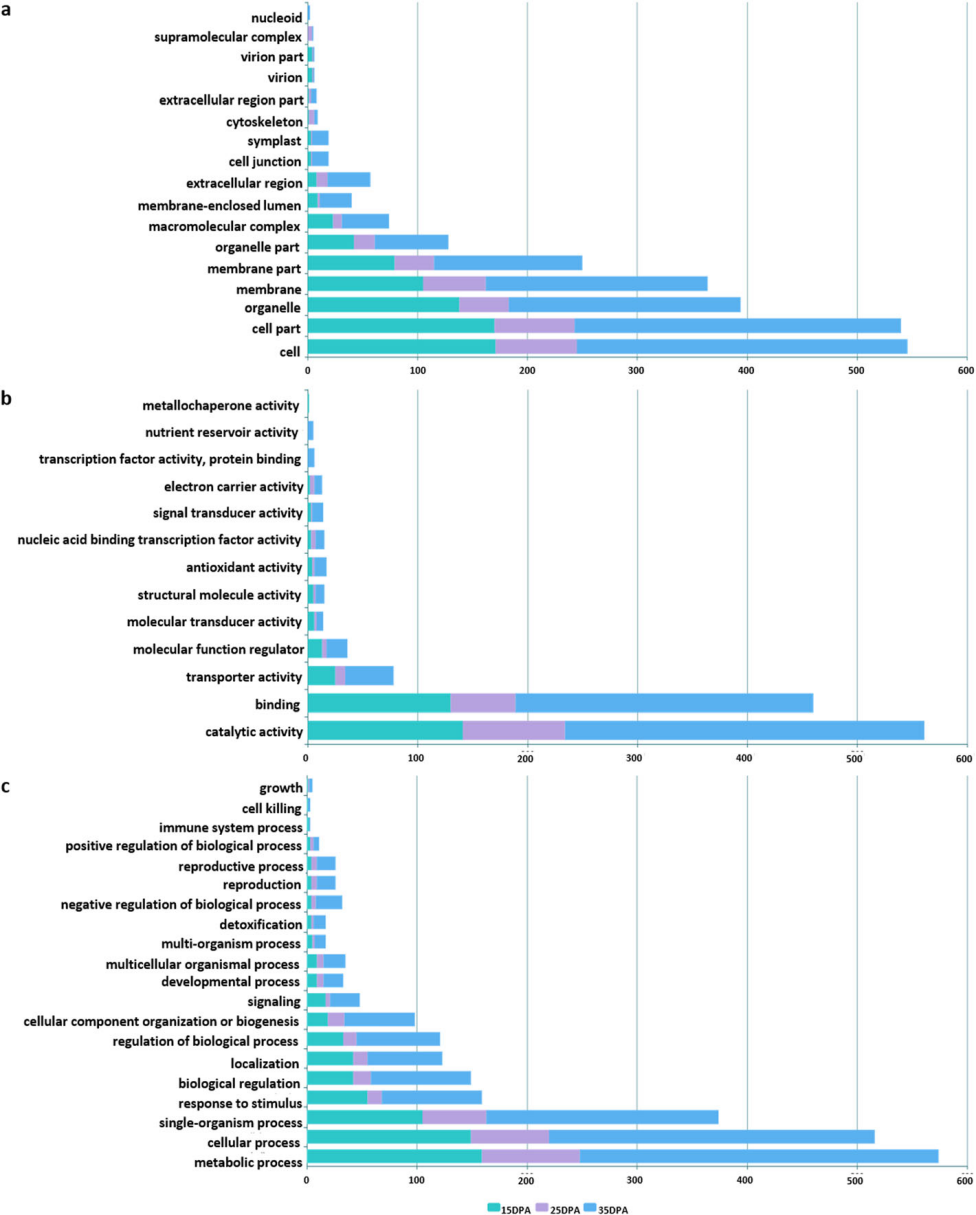
Gene ontology assignment of differentially expressed genes (DEGs) in the near-isogenic lines. The unigenes were mapped to three main categories: **a** cellular component, **b** molecular function, and **c** biological process. The x-axis indicates the number of annotated DEGs. DPA = days post anthesis

Based on GO descriptions, DEGs were functionally categorized into three principal categories: cellular component, molecular function and biological process (Fig. 3).

Cell, cell part, organelle, membrane, and membrane part were the most common terms in the cellular component category. Catalytic activity, binding, and transporter activity were the most abundant terms in the molecular function category at all three time-points. Most of the genes associated with the GO terms in the biological process category were in the subcategories of metabolic process, cellular process, and single organism process. Notably, all three GO categories had similar numbers of upregulated and downregulated genes in each of these categories at 15 and 25 DPA. However, at 35 DPA, both NIL pairs had considerably more upregulated genes than downregulated genes.

**Fig. 4:**
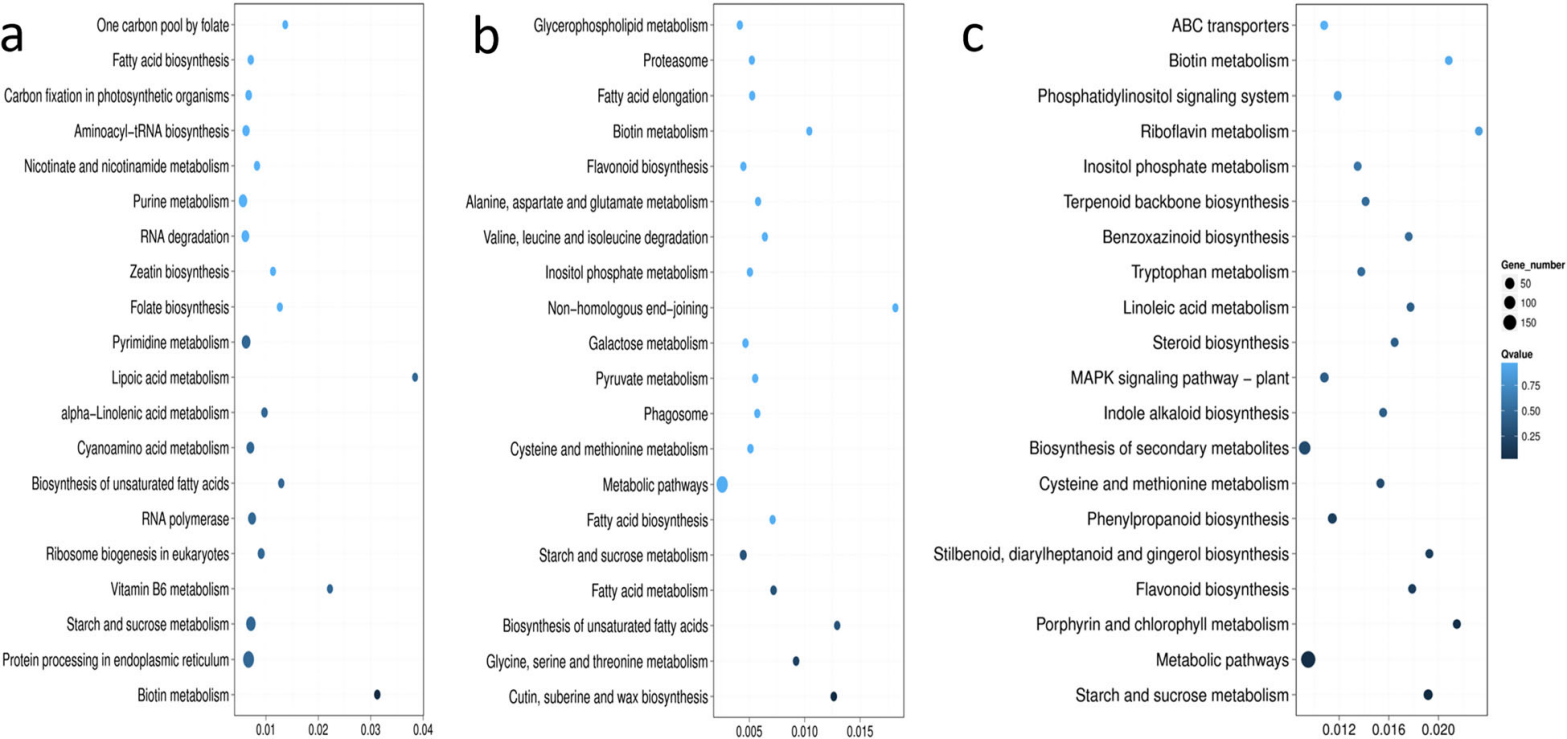
Pathway enrichment of differentially expressed genes (DEGs) in the near-isogenic lines at **a** 15 DPA, **b** 25 DPA and **c** 35 DPA. The x-axis indicates the rich factor. DPA = days post anthesis

Pathway enrichment analysis was performed to investigate related biological pathways that differed between isolines (Fig. 4, Table 2). DEGs across time-points in both NIL pairs were assigned to different pathways belonging to five major categories - cellular processes, environmental information processing, genetic information processing, metabolism and organismal systems. Among them, metabolism was the most enriched pathway in the DEGs, with more downregulated genes than upregulated genes to varying degrees across the three time-points.

**Table 2:** Candidate genes and their expression profiles as revealed by RNA-seq

Gene ID	DEG at time point	Physical location	Gene annotation	Pathway	Expression details^a^
***TraesCS3A01G461400***	15, 25, 35 DPA	697,281,350 − 697,284,916	Fork head transcription factor 1	Purine metabolism	Up regulated (log2 = 5.70)
***TraesCS3A01G462000***	15, 25, 35 DPA	697,471,602 −697,474,693	B3 domain-containing transcription factor	Aminoacyl-tRNA biosynthesis	Up regulated (log2 = 1.43)
***TraesCS3A01G466700***	15, 25, 35 DPA	700,562,650 −700,564,244	Hydroxyethylthiazole kinase	Thiamine metabolism	Down regulated (log2 = 5.19)
***TraesCS3A01G459200***	15, 25 DPA	695,744,627 - 695,745,649	Leucine-rich repeat receptor-like protein kinase family protein	Fatty acid biosynthesis; starch and sucrose metabolism; phenylpropanoid biosynthesis; biosynthesis of secondary metabolites	Up regulated (log2=2.14)
*TraesCS3A01G245000*	25, 35 DPA	458,679,119 − 458,682,490	Receptor kinase	Plant hormone signal transduction	Down regulated (log2 =4.00)
*TraesCS3A01G225100*	15 DPA	421,719,543 − 421,722,510	S-type anion channel	MAPK signalling pathway - plant	Up regulated (log2 =1.74)

Genes in bold fell into the QTL (*QPhs.ccsu-3A.1*) marker interval of Xwmc153 and Xgwm155, with a physical position within 484,402,604–702,961,948 bp

*DPA* Days post-anthesis, *Upregulated* Genes expressed higher in resistant isolines, *Downregulated* Genes expressed higher in susceptible isolines, *log2* Mean log2 ratio fold-change of the DEG in both NIL pairs

^a^The upregulation and downregulation of DEGs is based on comparisons of resistant to susceptible isolines

**Fig. 5:**
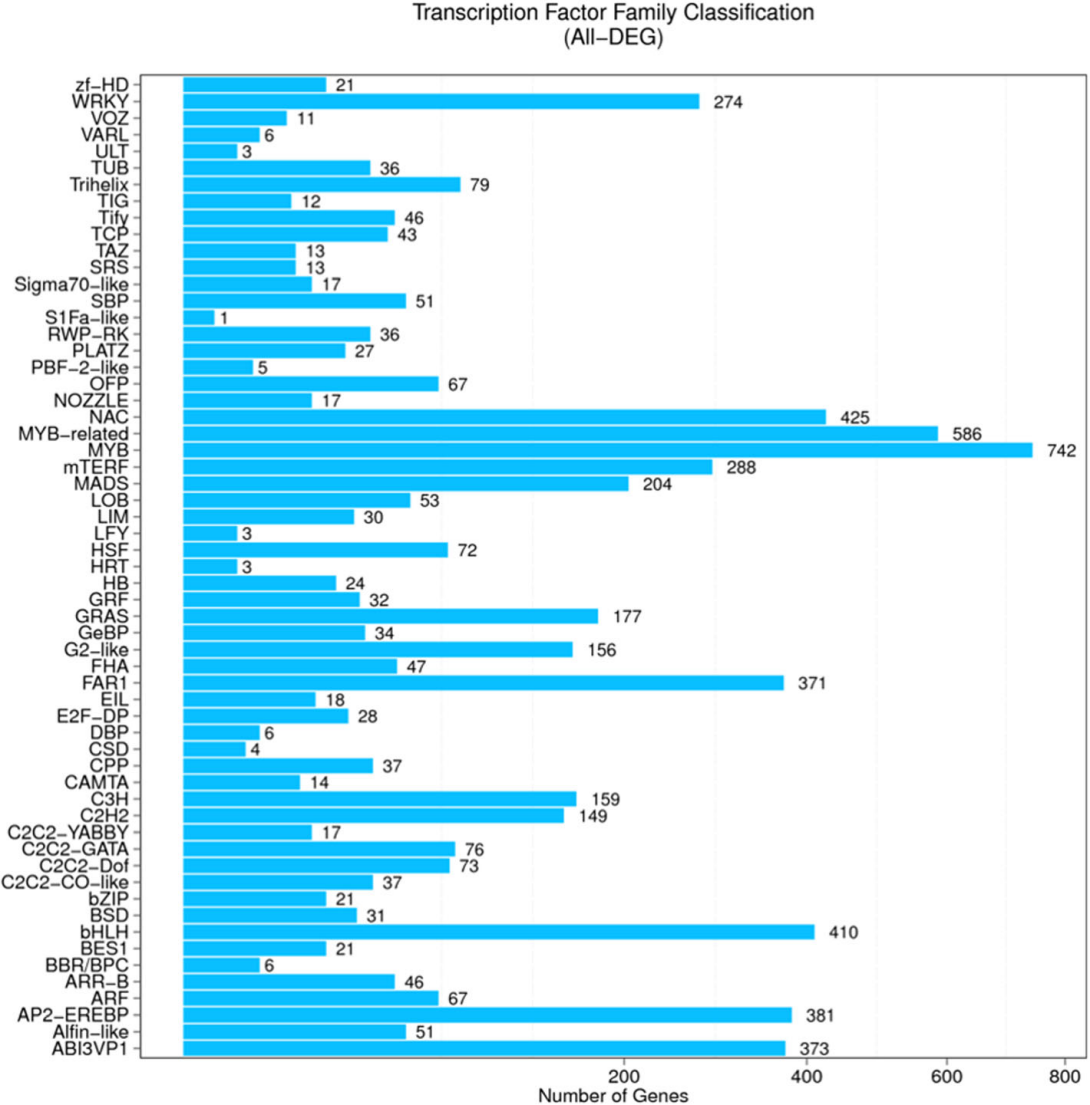
Distribution of differentially expressed genes (DEGs) in different transcription factor (TF) families in the whole transcriptome

Transcription factors (TFs) play a vital role as molecular switches controlling the expression of certain genes and in turn regulating plant growth and development under certain environmental conditions. Extensive database searches of all the DEGs at all the time-points in all the isolines predicted 6050 differentially expressed TFs which were grouped into 59 families (Fig. 5). The MYB and MYB-related TFs had the most genes (742 and 586 genes respectively), followed by NAC (425) and bHLH (410). However, none of the four extensively expressed TFs showed consistent DEG patterns in the NIL pairs at different time-points.

DEGs between the isolines that were common in both NIL pairs at each DAP were scrutinized; those with known functions related to PHS regulation pathways, such as plant hormone signal transduction and MAPK signaling were considered potential candidate genes. Based on this, three other genes *TraesCS3A01G459200*, *TraesCS3A01G245000* and *TraesCS3A01G225100* located on chromosome arm 3AL were identified as candidate genes (Table 2).

### SNP and indel markers polymorphic between the ‘R’ and ‘S’ isolines

The SNPs and indels showing consistent distinguishable genotypes between the isolines in both NIL pairs were detected. Five SNPs and 11 indels were located within or very close to the targeted 3AL QTL interval. Among them, six variants (three SNPs and three indels) occurred within their associated genes, with five falling in the gene exons and one in the untranslated region (UTR). Although other variants did not overlap any genes annotated in the reference genome, they showed short distances to their closest genes, with marker-gene distances ranging from 49 to 73,788 bp (Table 3).

**Table 3:** SNP and indel variants between resistant and susceptible NILs within the targeted 3AL QTL interval

No.	Variant type	Variant (R/S)	Variant in RefV1.0	Variant physical position	Associated gene	Gene function	Note
1	SNP	C/G	C	697,456,690	*TraesCS3A01G461800*	F-box domain containing protein	Within gene exon
2	SNP	G/A	A	702,901,184	*TraesCS3A01G471100*	Salicylate O-methyltransferase	2629 bp away from the gene
3	SNP	C/A	C	698,019,550	*TraesCS3A01G463700*	Transmembrane protein 45B	Within gene exon
4	SNP	T/C	T	697,250,101	*TraesCS3A01G461100*	Succinate dehydrogenase subunit 4	49 bp away from the gene
5	SNP	C/G	C	697,561,360	*TraesCS3A01G462400*	2-oxoglutarate (2OG) and Fe (II)-dependent oxygenase superfamily protein	Within gene exon
6	Indel	GT/−	G	695,515,689	*TraesCS3A01G458300*	F-box-like protein	Within gene exon
7	Indel	GATATTC/−	G	695,516,298	*“*	“	Within gene exon
8	Indel	C/CT	C	695,585,583	*TraesCS3A01G458400*	Nucleotide-diphospho-sugar transferases superfamily protein	52,358 bp away from the gene
9	Indel	A/−	AG	703,016,114	*TraesCS3A01G471400*	Basic 7S globulin 2	194 bp away from the gene
10	Indel	G/−	GA	703,016,906	*“*	“	986 bp away from the gene
11	Indel	C/−	CCG	688,767,420	*TraesCS3A01G449300*	Auxin response factor	73,788 bp away from the gene
12	Indel	CAA/−	C	688,767,424	*“*	“	73,784 bp away from the gene
13	Indel	AT/−	A	688,768,940	*“*	“	43,715 bp away from the gene
14	Indel	C/−	CGAG	700,588,842	*TraesCS3A01G467100*	UV-stimulated scaffold protein A-like protein	2576 bp away from the gene
15	Indel	G/−	GATGCGGTC	695,658,402	*TraesCS3A01G609800LC*	Glucose-1-phosphate adenylyltransferase	Within gene UTR
16	Indel	−/G	GC	686,681,832	*TraesCS3A01G592100LC*	Retrovirus-related Pol polyprotein from transposon opus	3374 bp away from the gene

*R* Resistant isoline, *S* susceptible isoline

*“*, value is the same as above

Twelve genes were associated with the SNPs and indels (Table 3). Of these, eight genes showed different expressions between either isolines or time-points. Apart from *TraesCS3A01G449300* functioning as an auxin response factor, no other gene was related to the hormone signaling pathway. For *TraesCS3A01G449300,* no expression difference was detected between ‘R’ and ‘S’ isolines in either of the NIL pairs (Table S1).

### qRT-PCR validation of candidate genes

To confirm the results of the RNA-seq, the six candidate genes were selected for qRT-PCR assays. Relative expressions of *TraesCS3A01G461400*, *TraesCS3A01G462000* and *TraesCS3A01G466700* differed significantly between the ‘R’ and ‘S’ isolines at all time-points, while that of *TraesCS3A01G245000* differed significantly at 25 DPA and 35 DPA, and *TraesCS3A01G225100* differed significantly at 15 DPA. Notably, at all time-points, the relative expression level of *TraesCS3A01G461400* differed about two-fold between the isolines. All six genes showed consistent expression patterns with those obtained from the RNA-seq analysis (Table 4). This is a strong indication of the reliability of the RNA-seq conducted in this study.

**Table 4:** qRT-PCR primers and results

Gene	Direction	Sequence (5′→3′)	NIL	Mean relative expression at 15 DPA	Mean relative expression at 25 DPA	Mean relative expression at 35 DPA
*TraesCS3A01G461400*	Forward	TTGCCGCTACTCAACAAGCC	R	16.20**	12.99**	11.09**
	Reverse	TCTGTTGTCTTCGCCGACT	S	8.47	7.95	5.25
*TraesCS3A01G462000*	Forward	CATATCATTCGCAAATCCCT	R	8.97*	5.76*	4.23*
	Reverse	TCTTCTGGCCTTACCCAA	S	7.09	4.54	3.01
*TraesCS3A01G466700*	Forward	CCCTGCAAGCCTCGAAAGCAT	R	5.36*	5.07*	4.25*
	Reverse	AACCTGCTCGCCATCCGTGA	S	5.98	5.81	5.03
*TraesCS3A01G459200*	Forward	CTTCCTTGAGCTCTCCTTCAAC	R	6.71*	6.44*	5.83
	Reverse	GCAGACTTGCTGAGGAACAT	S	5.58	5.50	5.33
*TraesCS3A01G245000*	Forward	TGGGACTCTTGTTCTCGCTCT	R	3.42	3.72*	2.98*
	Reverse	TCATCGTGCCAGAATGTGACC	S	3.41	4.48	3.58
*TraesCS3A01G225100*	Forward	ACGTTCAGTTTCCACACGGGTC	R	12.73**	9.26	7.72
	Reverse	GCCCAACTCCATACCGATCGAGA	S	9.71	9.47	7.49
*Actin*	Forward	CTCCCTCACAACAACCGC				
	Reverse	TACCAGAACTTCCATACCAAC				

*, significant difference (*p* ≤ 0.05); **, highly significant difference (*p* ≤ 0.01) between ‘*R*’ (resistant) and ‘*S*’ (susceptible) isolines, based on t-test; mean relative expression is the mean of NIL1 and NIL2; expression data are shown in ΔCt values, calculated by subtracting the Ct number of the reference gene (*Actin*) from that of the target gene

## Discussion

### Candidate genes underlying the major 3AL QTL responsible for PHS resistance

Six candidate genes underlying *QPhs.ccsu-3A.1* responsible for PHS resistance were identified in this study, based on their RNA-seq DEG profiles and qRT-PCR validations. Among them, *TraesCS3A01G461400* with a forkhead TF function is the most prominent candidate with highly significant differences in expression between ‘R’ and ‘S’ isolines in both its RNA-seq DEG profile and qRT-PCR expression analysis. Forkhead TFs are a family containing a DNA-binding domain known as the forkhead box (FOX). FOX is evolutionarily conserved in eukaryotic organisms and a crucial regulator of embryonic development, which is affected by hormone signaling [33, 34]. In contrast to the highly conserved FOX domain, forkhead TF proteins are highly divergent in other parts of their sequences [35]. In humans, forkhead TFs modulate signaling pathways [36], and can be a direct target of hormonal medications such as progestin to inhibit epithelial cell growth [37]. In insects, forkhead TFs regulate hormone-mediated signaling, affecting carbohydrate, amino acid and fatty acid metabolism, and the phosphatidylinositol 3-kinase/protein kinase B signaling pathway [38]. In plants, forkhead-associated domains mediate interactions with receptor-like kinases, which in turn regulate signaling pathways involved in growth and pathogen responses; two well-studied genes, *KAPP* (encodes a kinase-associated protein phosphatase that functions in the internalization of somatic embryogenesis receptor kinase 1) and *ABA1* (encodes a zeaxanthin epoxidase that functions in the ABA biosynthesis pathway), are among the 15 identified Arabidopsis genes containing forkhead-associated domains [39, 40]. Furthermore, *TraesCS3A01G461400* is involved in purine metabolism pathway which can play a role in activation of ABA metabolism [41, 42].

*TraesCS3A01G462000* encodes a B3 domain-containing TF; its family controls embryo development and seed maturation by modulating ABA and GA metabolism [43]. B3 TFs are considered specific to photosynthetic eukaryotes [44]. *Vp-1* is the first plant gene identified in maize that encodes a B3 type TF, which is a key component of the ABA signaling pathway during seed maturation in other cereals. *Vp-1* and its orthologous genes are associated with the activation of genes encoding seed storage proteins, late embryogenesis abundant enzymes, and anthocyanin biosynthesis enzymes, as well as the repression of post-germination genes for reserve mobilization, e.g. α-amylases and protease [45].

*TraesCS3A01G461400* and *TraesCS3A01G462000* are both TF genes. TFs play important roles in plant growth, development, and responses to environmental stress [46]. The interaction of sequence-specific TFs with target sites near their regulated genes is a central mechanism of gene expression regulation by which organisms develop and interact with their environment [47]. Both of the TF genes identified in this study are related to hormone signaling pathways, and showed significantly higher expression in ‘R’ than ‘S’ isolines at all time-points, suggesting that the more active regulation of gene transcriptions in the ‘R’ isolines might contribute to its PHS resistance phenotype.

Interestingly, two other candidate genes *TraesCS3A01G459200* and *TraesCS3A01G245000* function as receptor-like kinases (RLKs). RLKs are surface localized, transmembrane receptors that regulate a variety of signaling pathways [48, 49]; some have interactions with forkhead-associated domains, such as KAPP [40, 50]. Gene *TraesCS3A01G459200* is involved in many metabolism pathways including fatty acid biosynthesis, starch and sucrose metabolism, phenylpropanoid biosynthesis, and biosynthesis of secondary metabolites (Table 2), in which phenylpropanoid metabolism has been reported to relate to primary seed dormancy in Arabidopsis [51]. Gene *TraesCS3A01G245000* was directly involved in the plant hormone signal transduction pathway by KEGG enrichment analysis. Leucine-rich repeat (LRR) RLK, encoded by *TraesCS3A01G459200,* plays an important role in ABA signal transduction in Arabidopsis; it is upregulated by ABA and its loss of function results in ABA insensitivity in seed germination [52]. In this study, LRR RLK had significantly higher expression in ‘R’ than ‘S’ isolines at 15 and 25 DPA, but there was no difference at 35 DPA between the isolines as the gene was downregulated in ‘R’ isolines at 35/15 (Table S1), indicating a higher ABA content in ‘R’ isolines at the early stages.

*TraesCS3A01G466700* encodes hydroxyethylthiazole kinase which participates in thiamine biosynthesis pathway [53]. Taking part in glycometabolism, thiamine has a fundamental role in energy metabolism and serves as an energy reserve for seed germination [54]. Golda et al. [54] found that both cereal and legume seeds lost a significant part of their thiamine reserves during germination. Neumann et al. [55] reported that seeds treated with thiamine significantly increased their germination rate in legume *Phasenius vulgaris*. The gene showed significantly lower expression in ‘R’ than ‘S’ isolines at all time-points in this study, implying that a lower thiamine reserve exists in ‘R’ isolines, which may not favor germination.

*TraesCS3A01G225100* functions in the S-type anion channel activity which is required for ABA-induced gene expression [56]. KEGG enrichment assigned the gene to plant MAPK signaling pathway. The MAPK module directly responds to ABA, or interacts with MKK3; for example, MAP3K16 is the negative regulator of ABA response (ABR1), and MAP3K17/18-MKK3-MPK1/2/7/14 responds to ABA, senescence and dormancy in Arabidopsis [57, 58]. MKK3 contains an NTF2 domain and its primary gene structure is highly conserved during evolution [59]. Torada et al. [21] reported that *MKK3* was the causal gene underlying the major 4AL QTL responsible for seed dormancy in wheat. In this study, *TraesCS3A01G225100*showed significant higher expression in ‘R’ than ‘S’ isolines at 15 DPA only, indicating that the gene mainly plays a regulatory role in response to ABA at an early stage of seed development.

### SNP and indel markers distinguishable between the ‘R’ and ‘S’ isolines

Ten of the 16 SNP or indel variants between the ‘R’ and ‘S’ isolines did not overlap any annotated genes in RefV1.0. However, as these variants were identified based on the RNA-seq profiles, they should have been within transcribed genes of the tested cultivars/lines. This response may be due to: 1) large structural variations between the genomes of the reference cultivar Chinese Spring (CS) and the tested cultivars/lines; or 2) CS does not contain certain genes that exist in other cultivars, for example, *Ppd-B1* and *Vrn-A1* alleles were not present in CS [60].

Gene *TraesCS3A01G449300*, associated with one of the indel markers, functions as an auxin response factor (ARF). Auxin recruits ARFs to control seed dormancy in Arabidopsis through stimulation of ABA signaling by inducing ARF-mediated ABI3 activation [61]. Auxin is involved in the transition from seed dormancy to germination, which promotes seed dormancy and inhibits seed germination [62]. Although its gene expression did not differ between the contrasting isolines, its location within the targeted major QTL suggestes it is involved in the regulation pathway by interacting with other signal transduction genes sitting in that particular genomic region.

Within the QTL interval, there is another noteworthy genomic region from 622,277,558 to 622,901,141 bp, where a block of consecutive genes exist that are related to basic leucine zipper (bZIP) TFs and ABI5s. BZIP TFs are activated by ABA-mediated signalosome and bind to specific cis-acting sequences called abscisic-acid-responsive elements (ABREs) or GC-rich coupling elements, thereby influencing the expression of their target downstream genes [63]. ABIs are involved in ABA signaling, some of which are TFs; ABI5 is one of the six classes of such TFs that have been identified, and is essential for ABA- or seed-specific gene expression [64]. Transcriptional repressions of ABI5 are associated with reduced seed sensitivity to ABA which results in the switch from dormancy to germination in wheat seed [65]. No consistent expression difference between the contrasting isolines was found in these genes at the three time-points investigated in this study (Table S1). However, as the genes function in the core ABA signaling and appear in a cluster within the targeted QTL interval, it implies the possibility that multiple genes and gene-interactions could underlie the QTL responsible for PHS resistance.

The targeted QTL *QPhs.ccsu-3A.1*, explaining up to 78.03% of the phenotypic variation [26], could be exploited as a key locus for marker-assisted selection. Many genes in the QTL region, including the identified candidate genes, SNP/indel marker associated genes and physically clustered genes, are involved in hormone perception and signal transduction, which further demonstrates the significance of the locus in the regulation and control of PHS resistance. For future study, allele characterizations can be conducted in other genotypes known to have the QTL, and transgenic approach can be utilized for functional test of the candidate genes.

## Conclusions

Transcriptomic profiling of NILs targeting a major 3AL QTL *QPhs.ccsu-3A.1* responsible for PHS resistance revealed six candidate genes related to hormone signaling and energy metabolism. Sixteen SNP or indel markers within the QTL interval showed consistent distinguishable alleles between the ‘R’ and ‘S’ isolines contrasting in PHS performance. The targeted QTL was confirmed as a key genomic region for seed dormancy and PHS resistance as it contained many core genes involved in the ABA signaling pathway, some of which showed significant differences in expression between the contrasting isolines. The identified candidate genes and SNP/indel markers in this study are valuable for understanding the mechanism of PHS resistance and for marker-assisted breeding of the trait in wheat.

## Methods

### Plant material and tissue sampling

In a previous study, we generated a set of NILs using the heterogeneous inbred family method targeting the 3AL QTL [27]. Formal identification of the plant materials have been done through genotyping and phenotyping [27] by all the authors who are experts in wheat. Two pairs of NILs (each pair was derived from the single seed descent of an F2 individual) with significantly contrasting PHS performance between the isolines were used for RNA-seq in this study (Fig. 2). Both NIL pairs were developed from the population of ‘Chara/DM5637B*8’ and named as ‘NIL pair 1R & 1S’ and ‘NIL pair 2R & 2S’ in this study, matching NIL_PHSR3AL_3R & 3S and NIL_PHSR3AL_6R & 6S in the previous study, respectively [27]. ‘R’ indicates isolines carrying the resistant allele, and ‘S’ is for those with the susceptible allele. The seeds of parent ‘Chara’ were obtained from Australian Grains Genebank, Horsham, Victoria, Australia with a deposition number of AUS30031, and the seeds of parent ‘DM5637B*8’ were obtained from InterGrain Pty Ltd., Australia. The seeds of the NILs used in this study are kept at the University of Western Australia Wheat Seed Collection with deposit numbers of UWANILTa-N11 (NIL_PHSR3AL_3R), UWANILTa-N12 (NIL_PHSR3AL_3S), UWANILTa-N17 (NIL_PHSR3AL_6R) and UWANILTa-N18 (NIL_PHSR3AL_6S).

The NILs were grown with three biological replicates for each isoline in the glasshouse of The University of Western Australia in Perth, Western Australia. The plant growth condition and phenotyping methods were the same as described in Wang et al. [27]. The flowering date was recorded for each spike. Five kernels at 15 DPA and 25 DPA and three kernels at 35 DPA from each isoline in each replicate were randomly collected, frozen immediately in liquid nitrogen and stored at − 80 °C for RNA extraction.

### RNA extraction, library construction and Illumina sequencing

Total RNA was extracted from 36 samples (4 genotypes × 3 time-points × 3 replicates) using RNeasy Plus Plant Mini Kit (Qiagen) with the treatment of DNase, following the manufacturer’s instructions. The yield and purity of the extracted RNA were assessed by NanoDrop 2000 (Thermo Fisher Scientific Inc., Australia), and the integrity was checked by 1% (w/v) denatured gel electrophoresis and Agilent 2100 Bioanalyzer (Agilent Technologies Inc., USA). The qualified and quantified RNA samples were sequenced at the Beijing Genomics Institute (BGI), China. The BGI protocol for cDNA synthesis, 150 bp paired-end sequencing and raw data filtering were the same as described in Mia et al. [30]. Clean data were generated as FastQ files, and Q20, Q30 and GC contents were calculated. Downstream analyses were performed on these clean data, which are available at the National Centre for Biotechnology Information (NCBI) website with the SRA accession number of PRJNA554312 (https://www.ncbi.nlm.nih.gov/sra/PRJNA554312).

### Sequence data analysis and DEG identification

High-quality reads were mapped to the bread wheat reference genome sequence, international wheat genome sequence consortium (IWGSC) RefSeq V1.0 (https://wheat-urgi.versailles.inra.fr/) [66], using HISAT2 v2.0.4 [67]. Aligning of the reads to the reference sequence was done by Bowtie2 [68]. Gene expression level were calculated using RSEM v1.2.12 [69] with default parameters. DEGs were identified with DEGseq according to Wang et al. [70] with the parameters as described in Mia et al. [30]. Up- and down-regulations of DEGs between the isolines were based on the comparison of ‘R’ isoline to ‘S’ isoline, i.e., if a gene expression in ‘R’ isoline was higher or lower than that in ‘S’ isoline, it was considered upregulated or downregulated, respectively.

### Functional annotations, gene ontology and pathway analyses

Gene ontology (GO) and functional enrichment of the selected DEGs were performed using a hypergeometric test (phyper); those with a false discovery rate (FDR) ≤ 0.01 were considered as significantly enriched. KEGG annotation was the same as described in Mia et al. [30]. To identify TF encoding genes from the DEGs, Getorf tool [71] was used to find the open reading frame (ORF) of each DEG. The ORFs were then aligned to TF domains from PlnTFDB using hmmsearch [72] to identify TF encoding genes from the selected DEGs.

### Discovery of SNP and indel markers

To find the SNP and indel variants, all clean reads of the transcripts were mapped to the reference genome sequence of IWGSC RefV1.0 (https://wheat-urgi.versailles.inra.fr/) using Bowtie2 [68]. The SAM tools package was used for calling SNP and indel variants. Variants on the 3A chromosome, especially those within the marker interval of Xwmc153 and Xgwm155, were detected.

### Validation of DEGs using quantitative RT-PCR (qRT-PCR) analysis

The candidate genes identified in this study were selected to run qRT-PCR to validate the RNA-seq results. The cDNAs were synthesized using SensiFast cDNA Synthesis Kit (Bioline Australia) with the manufacturer’s protocol. The qRT-PCR was performed on an ABI 7500 Fast system using SensiFAST SYBR kit (Bioline Australia). Gene-specific primers were designed using Primer Premier 5.0 software, and the wheat actin gene was used as an endogenous control for normalization between samples. Three biological replicates were used for each isoline of the two pairs of NILs at the three time-points. For qRT-PCR, cDNA from all biological samples was run in triplicate [73]. Amplification was conducted in a 20 μl reaction mix containing 10 μl of 2 × SensiFAST SYBR Lo-ROX mix, 0.8 μl of 10 μM each forward and reverse primer and 100 ng cDNA, with the following cycling protocol: 1 cycle of 95 °C for 2 min, 40 cycles of 95 °C for 5 s and 60 °C for 30 s. Relative gene expression was calculated using the comparative Ct method [74]. A two sample t-test was used to compare the expression differences between the means of ‘R’ and ‘S’ isolines at different DPAs.

## Supplementary Information

**Additional file 1: Table S1.** DEGs with noteworthy features and located within the targeted QTL marker interval of Xwmc153 and Xgwm155 (physical position of 484,402,604–702,961,948 bp).

## Data Availability

The datasets generated and/or analysed during the current study are available in the National Center for Biotechnology Information (NCBI) website with the SRA accession number of PRJNA554312 (https://www.ncbi.nlm.nih.gov/sra/PRJNA554312). The plant materials (seeds) are kept at the University of Western Australia Wheat Seed Collection.

## Abbreviations

ABA: Abscisic acid
ABI: ABA insensitive
DEG: Differentially expressed genes
FDR: False discovery rate
FOX: Forkhead box
GA: Gibberellic acid
GO: Gene ontology
NIL: Near-isogenic line
ORF: Open reading frame
PHS: Pre-harvest sprouting
PIF: Phytochrome interacting factor
QTL: Quantitative trait locus/loci
RNA-seq: RNA sequencing
SNP: Single nucleotide polymorphism
TF: Transcription factors
